# Sleep dysfunction associated with worse chemotherapy-induced peripheral neurotoxicity functional outcomes

**DOI:** 10.1007/s00520-023-08245-w

**Published:** 2023-12-20

**Authors:** Fawaz Mayez Mahfouz, Tiffany Li, Masarra Joda, Michelle Harrison, Lisa G. Horvath, Peter Grimison, Tracy King, Gavin Marx, David Goldstein, Susanna B. Park

**Affiliations:** 1 Brain and Mind Centre, The University of Sydney, Camperdown, NSW 2050 Australia; 2 Chris O’Brien Lifehouse, Camperdown, NSW 2050 Australia; 3 Sydney Medical School, The University of Sydney, Camperdown, NSW 2050 Australia; 4 Royal Prince Alfred Hospital, Camperdown, NSW 2050 Australia; 5 Cancer Nursing Research Unit, The University of Sydney, Camperdown, NSW 2050 Australia; 6 Institute of Haematology, Royal Prince Alfred Hospital, Camperdown, NSW 2050 Australia; 7 Sydney Adventist Hospital, Wahroonga, NSW 2076 Australia; 8 Prince of Wales Clinical School, Faculty of Medicine & Health, UNSW Sydney, Randwick, NSW 2031 Australia; 9 Department of Medical Oncology, Prince of Wales Hospital, Randwick, NSW 2031 Australia

**Keywords:** Chemotherapy-induced peripheral neurotoxicity (CIPN), Sleep impairment, Sleep quality, Insomnia, Cancer survivors

## Abstract

**Purpose:**

Sleep problems are commonly reported by cancer survivors; however, knowledge of the impact of chemotherapy-induced peripheral neurotoxicity (CIPN) on sleep quality remains limited. In this study, we explored the impact of CIPN on sleep quality, as well as identified clinical characteristics associated with poor sleep quality.

**Methods:**

Participants were assessed cross-sectionally post-neurotoxic chemotherapy. CIPN severity was graded using a range of questionnaires that assessed CIPN severity and quality of life, as well as neurological grading scales. Sleep quality was assessed using a self-rated questionnaire (Pittsburgh Sleep Quality Index, PSQI). Participants with poor sleep quality were further grouped according to whether sleep impairment was due to CIPN or other factors.

**Results:**

Among 77 participants who reported CIPN, 75% (*n* = 58) reported poor sleep quality. Of those, 41% (*n* = 24) reported CIPN as contributing to sleep impairment, while 59% (*n* = 34) reported other causes. Participants with CIPN-induced sleep impairments had higher CIPN severity across all outcome measures, as well as greater neuropathic pain (all *p* < 0.05). Furthermore, participants with CIPN-induced sleep impairments reported worse impact of neuropathy on physical and social functioning, as well as emotional well-being (all *p* < 0.05).

**Conclusions:**

Participants with CIPN-induced poor sleep quality reported worse scores across all CIPN severity measures. This emphasises the negative impacts of CIPN symptoms on quality of life of chemotherapy-treated patients and highlights the importance of sleep quality assessment in cancer survivors.

**Supplementary Information:**

The online version contains supplementary material available at 10.1007/s00520-023-08245-w.

## Introduction

Sleep problems are prevalent in cancer patients, but often overlooked. Approximately 25 to 60% of chemotherapy-treated patients report poor sleep quality, particularly experiences of sleep disturbance, early awakening, difficulty falling asleep, and excessive sleepiness during the day [1, 2]. More so, several studies have reported the association between poor sleep and fatigue, anxiety, and depression in cancer survivors [3–5]. There are multiple causes of sleep dysfunction in chemotherapy-treated patients, including cancer-related symptoms and treatment side effects such as pain, nausea, altered bowel and bladder function, and mood disturbance [6].

One common consequence of chemotherapy treatment is chemotherapy-induced peripheral neurotoxicity (CIPN), which produces symptoms of numbness, tingling, neuropathic pain, and functional loss, which reduces quality of life of cancer patients [7]. Worse CIPN severity has been associated with increased sleep disturbance and depression in colorectal cancer survivors 1 to 7 years post-chemotherapy [8]. Similarly, a longitudinal study of colorectal cancer patients demonstrated that the development of sensory or motor peripheral neuropathy was significantly associated with poor sleep quality with no improvements at 1 to 2 years post-cancer diagnosis [9].

There may be multiple contributors to poor sleep quality in patients with CIPN. Neuropathic pain has been reported to closely associate with declining sleep quality status in chemotherapy-treated patients [10, 11]. Accordingly, patients with painful CIPN may be at higher risk of developing anxiety, depression, and sleep disturbance [12, 13]. In a cohort of 501 breast cancer patients, the occurrence of severe neuropathic pain was associated with a deteriorating global sleep quality from baseline to 1-year follow-up, particularly shorter sleep duration, increased use of sleep medication, and trouble staying awake in social events and a lack of enthusiasm to get things done [14]. However, there is a lack of understanding of the specific impact of CIPN, including non-painful symptoms, on sleep quality in cancer survivors. Despite the potential interaction between CIPN symptoms and sleep quality, the impact of CIPN symptoms on sleep is not addressed in the majority of CIPN assessment tools. Limitations in our understanding of the relationship between CIPN phenotypes and sleep quality may result in a lack of appropriate interventions or supportive care to help manage sleep problems in this cohort.

Therefore, to help better understand the impact of CIPN on sleep quality [14] and guide appropriate intervention in this cohort, the aims of this study were to explore the impact of CIPN on sleep quality by identifying clinical characteristics of CIPN associated with sleep disturbance.

## Methods

### Participants

This study was approved by the Sydney Local Heath District (RPAH zone) Human Research Ethics Committee and conducted in accordance with the Declaration of Helsinki. Consenting participants with cancer who were ≥ 18 years and who had completed their neurotoxic chemotherapy treatment (including taxanes, platinum-based, bortezomib, vinca alkaloids, and thalidomide) were eligible for the cross-sectional study. Clinical data were retrieved from patient medical records. Informed consent was obtained from each participant.

### Sleep assessment: patient-reported outcome measures

Assessment of sleep quality and patterns were undertaken via the Pittsburgh Sleep Quality Index (PSQI). PSQI is a 19-item questionnaire comprised of seven subdomains: subjective sleep quality, sleep latency, sleep duration, sleep efficiency, sleep disturbance, the use of sleep medications, and daytime dysfunction over the past month. Each component has a 3-point score, with 0 indicating ‘no trouble on sleep during the past month’; 1, ‘trouble less than once a week’; 2, ‘trouble once or twice a week’; and 3, ‘trouble three or more times a week’. Each subdomain was broken down into components of varying severity and dysfunction, according to a publicly available algorithm. All seven components sum up to provide a global PSQI score ranging from 0 to 21, with higher scores indicating worse sleep quality [15].

Sleep disturbance was measured using the Patient-Reported Outcomes Measurement Information System (PROMIS-SD) 8-item short form (v. 1.0; 8a) [16]. It consists of 8-items that measure self-reported perceptions of sleep depth, restoration, and quality in the past week prior to testing. Each item has a 5-point Likert scale, and the sum of all 8-items generated a raw score, which was then converted to a standardised T-score according to the conversion tables published on the PROMIS website (nihpromis.org). Higher T-scores indicated greater sleep disturbances.

### Patient-reported outcome measures, clinical neuropathy assessment, and functional assessment

Assessment tools are briefly described below with further details available in supplementary methods.

The Chronic Acquired Polyneuropathy Patient-Reported Index (CAP-PRI) is a health-related quality of life (HRQoL) measure that were used to assess patient’s emotional well-being, pain severity, and social and physical functioning, including trouble sleeping due to neuropathy [17]. The European Organisation of Research and Treatment of Cancer Quality of Life Questionnaire-Core (EORTC-QLQ-CIPN20) was used to assess autonomic, motor, and sensory peripheral neuropathy symptoms [18]. The Patient-Reported Outcomes version of the Common Terminology Criteria for Adverse Events (PRO-CTCAE) was used to assess the severity and interference of the numbness and tingling in the hands and feet [19]. A modified version of the Pain Numeric Rating Scale (PNRS) was used for the assessment of the intensity of neuropathic pain experienced [20].

The severity of CIPN was clinically graded by research assistants using the National Cancer Institute Common Terminology Criteria for Adverse Events (NCI-CTCAE) sensory subscale Version 4.0 with CIPN severity graded on a scale from grade 0 = no CIPN to grade 4 = disabling. Total Neuropathy Score-clinical version (TNSc©, John Hopkins University) which comprised patient report, sensory, and neurological examination also graded CIPN severity [21–23]. Assessment of sensory acuity in the fingers of the dominant hand was undertaken by identifying the perception threshold for the Grating Orientation Task (GOT) and Von Frey monofilament task [24, 25]. Assessment of fine motor skills and manual dexterity was undertaken via time taken to complete the Grooved Pegboard Task [26]. Further details are in supplementary methods.

Neurophysiological measurements, including nerve conduction studies measuring sural and tibial nerve amplitudes of the lower limb as well as sensory and motor median nerve amplitudes of the upper limb, were undertaken, following methodologies as per previous studies [27].

### Participant classification

Participants with no CIPN (NCI-CTCAE grade 0) were excluded from further analysis of the effects of CIPN on sleep quality. The remaining participants were classified based on their global PSQI score. Participants who had a global PSQI score of 3 5 were placed in the ‘Poor Sleep Quality’ group, while those with a score of < 5 comprised the ‘Good Sleep Quality’ group, as per previous work [28].

Participants in the ‘Poor Sleep Quality’ group were further classified to determine the cause of sleep impairment according to their responses to the CAP-PRI item ‘do you have trouble sleeping due to your neuropathy’. Responses of ‘A little bit’ or ‘A lot’ were placed in the ‘CIPN-induced sleep impairments’ group, while a ‘Not at all’ response comprised the ‘Sleep impairment due to other factors’ group. Furthermore, factors causing sleep impairments in participants with poor sleep quality were identified by reporting percentage of participants with sleep dysfunction (‘less than once a week’ OR ‘once or twice a week’ OR ‘three or more times a week’) to each item of the sleep disturbance subdomain of the PSQI (Q5a to Q5j), as well as participant responses to the semi-structured interview question ‘do you have trouble sleeping due to neuropathy symptoms?’.

### Statistical analyses

SPSS Statistics Software V27 (IBM, Armonk, NY) was used for all analyses in this study. Normality of data was evaluated using the Shapiro–Wilk test. A *p*-value of > 0.05 highlights the normally distributed data which were presented as mean ± standard deviation (SD), while a *p*-value of < 0.05 highlights the non-normally distributed data which were presented as medians and interquartile range (IQR). The associations between sleep outcome measures, demographic characteristics, functional assessments, CIPN severity, and pain outcome measures were undertaken using Pearson’s or Spearman’s correlation coefficients, for normally and non-normally distributed data, respectively. Group comparisons were also investigated using Mann–Whitney *U*, independent sample *t*-tests, or chi-square tests.

## Results

### Demographic and clinical history

A total of 87 participants were assessed cross-sectionally post-neurotoxic chemotherapy treatment. Out of 87 participants, 11% (*n* = 10) reported no CIPN at the time of assessment and were excluded from further analyses as the focus of this analysis was on the impact of CIPN on sleep quality.

The remaining 89% (*n* = 77) reported CIPN. They had a mean age of 63.4 ± 11.3 years and were 13.0 (IQR = 21.0) months post-neurotoxic chemotherapy treatment completion. Of those, 68% (*n* = 52) were female participants, mostly diagnosed with gynaecological (29%, *n* = 38) or haematological cancers (20%, *n* = 26). Taxane (50%, *n* = 38), platinum-based agents (25%, *n* = 19), or bortezomib (23%, *n* = 18) were the most common chemotherapy types administered to participants (Table 1). Overall, 39% (*n* = 30 of 77) of participants graded with mild CIPN (NCI-CTCAE grade 1) while 61% (*n* = 47) were graded with moderate-to-severe CIPN (NCI-CTCAE grade ≥2).

**Table 1:** Demographic and clinical history of participants with good sleep quality vs poor sleep quality. Comparisons between both groups were performed using chi-square tests. Demographic characteristics were also compared between good and poor sleep quality groups using independent sample *t*-tests. *Indicates *p*-values using Mann–Whitney *U* tests. *p* < 0.05 was considered significant were bolded

Participants with CIPN (*n* = 77)	Good sleep quality (*n* = 19)		Poor sleep quality (*n* = 58)		Total (*n* = 77)		*p*-value
	*n*	%	*n*	%	*n*	%	
*Clinical characteristics*							
Female sex	11	58	41	71	52	68	0.3
*Cancer type*							
Breast	1	5	5	9	6	8	0.9
Gynaecological (cervical, endometrial, and ovarian)	9	48	20	35	29	38	
Haematological (myeloma and Hodgkin’s lymphoma)	5	26	15	26	20	26	
GI/colorectal and pancreatic	2	11	10	17	12	15	
Testicular and prostate	1	5	2	3	3	4	
Other	1	5	3	5	4	5	
Missing	0	0	3	5	3	4	
*Chemotherapy type*							
Taxane	9	48	29	50	38	50	0.2
Platinum-based	4	21	15	26	19	25	
Bortezomib	4	21	14	24	18	23	
Vincristine	1	5	0	0	1	1	
Thalidomide	1	5	0	0	1	1	
*Cancer stage*							
0	0	0	1	2	1	1	0.9
I	3	16	6	10	9	12	
II	3	16	11	19	14	18	
III	6	32	15	26	21	27	
IV	2	10	6	10	8	10	
No stage (non-solid tumours)	5	26	15	26	20	26	
Missing	0	0	4	7	4	5	
*Demographic characteristics*							
	Mean	SD	Mean	SD	Mean	SD	*p*-value
Age (years)	**68.3**	**11.8**	**61.7**	**10.7**	**63.4**	**11.3**	**0.02**
BMI (kg/m^2^)	25.3	4.4	26.4	5.5	26.2	5.3	0.5
Months since treatment completion; median (IQR)*	14.0	24.0	12.5	20.0	13.0	21.0	0.3

### Sleep quality profile in chemotherapy-treated patients

Of the 77 participants who reported CIPN, 75% (*n* = 58) reported poor sleep quality, while 25% (*n* = 19) reported good sleep quality (Table 1). All demographic and clinical information for participants is found in Table 1.

**Fig. 1:**
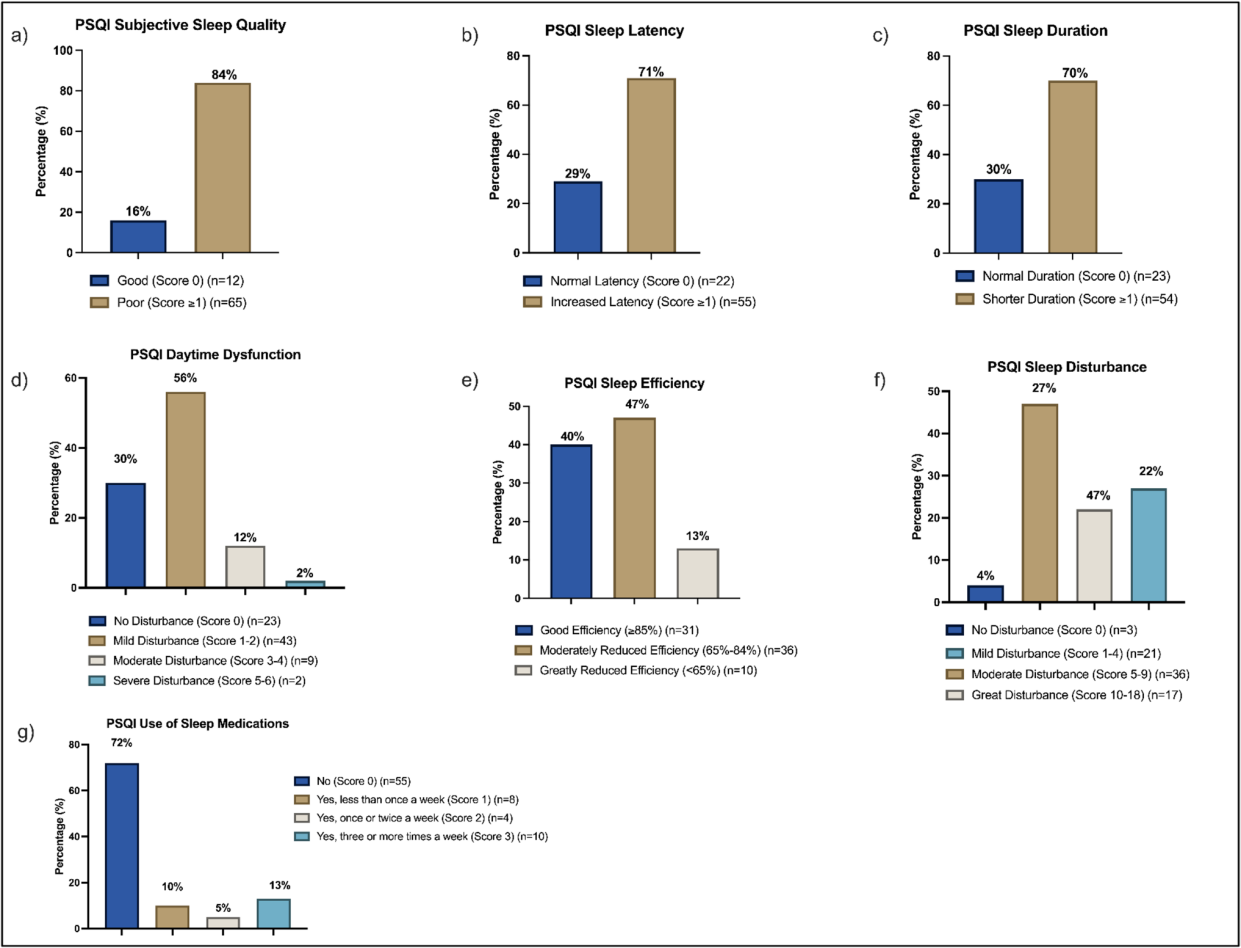
Percentage of all participants reporting sleep problems across PSQI subdomains, including **a** subjective sleep quality, **b** sleep latency, **c** sleep duration, **d** daytime dysfunction, **e** sleep efficiency, **f** sleep disturbance, and **g** the use of sleep medications in the past month prior to testing. Blue indicates normal responses and absence of dysfunction, while other colours indicate the presence of varying severity and dysfunction (indicated in key legend of each figure).

The outcomes of the subdomains of the self-reported sleep questionnaire (PSQI) are reported in Fig. 1. Overall, more than 70% of all participants reported poor subjective sleep quality (Fig. 1a), increased time taken to fall asleep (sleep latency) (Fig. 1b), shorter sleep duration (Fig. 1c), and mild-to-severe daytime dysfunction (Fig. 1d). More so, 60% of participants reported moderately to greatly reduced sleep efficiency (Fig. 1e), while 69% reported moderate-to-great sleep disturbance (Fig. 1f). However, only 30% of participants reported using sleep medications in the past month prior to testing (Fig. 1g).

Participants with poor sleep quality had greater impact on all components of their sleep, including worse subjective sleep quality, increased sleep latency, shorter sleep duration, greater sleep disturbance, and reduced sleep efficiency compared to participants with good sleep quality (all *p* ≤ 0.005) (Table 2).

**Table 2:** Comparison of neuropathy outcome measures between participants with good and poor sleep quality, using Mann–Whitney *U* tests. *p* < 0.05 was considered significant were bolded. *Indicates *p*-values using independent sample *t*-tests. Higher scores on CIPN outcome measures and function assessments, including lower amplitudes on neurophysiological measures, indicate worse impairment

Participants with CIPN (*n* = 77)	Good sleep quality (*n* = 19)		Poor sleep quality (*n* = 58)		*p*-value
	Median	IQR	Median	IQR	
*Functional assessments*					
Grating Orientation Task (GOT) threshold (mm)	3.9	3.1	3.3	1.2	0.3
Average pegboard time (s) (mean, SD)*	**84.2**	**16.3**	**70.9**	**14.7**	**0.002**
Von Frey threshold (mN)	0.4	0.7	0.4	0.5	0.8
*Sleep outcome measures*					
PSQI global score	**3.0**	**2.0**	**8.0**	**4.0**	** < 0.001**
PSQI subjective sleep quality	**1.0**	**1.0**	**1.0**	**1.0**	**< 0.001**
PSQI sleep latency	**1.0**	**2.0**	**2.0**	**3.0**	**0.005**
PSQI sleep duration	**0**	**1.0**	**1.0**	**1.0**	**< 0.001**
PSQI sleep efficiency (%) (mean, SD)*	**90.3**	**6.5**	**75.3**	**14.6**	** < 0.001**
PSQI sleep disturbance (mean, SD)*	**3.8**	**2.8**	**7.7**	**3.7**	** < 0.001**
PSQI use of sleep medications	**0**	**0**	**0**	**1.0**	**0.002**
PSQI daytime dysfunction	**0**	**1.0**	**1.0**	**1.0**	**0.005**
PROMIS sleep disturbance (T-score)	47.9	1.2	50.8	6.4	0.07
*CIPN outcome measures*					
Patient-Reported Outcome (PRO-CTCAE) Severity score	1.0	1.0	2.0	1.0	0.6
Patient-Reported Outcome (PRO-CTCAE) Interference score	0	1.0	1.0	1.0	0.1
Neurological Examination Score (TNSc)	5.0	3.0	4.0	4.0	0.5
Patient-Reported Outcome (EORTC-QLQ-CIPN20) score	12.3	10.5	15.3	14.0	0.2
Health-Related Quality of Life Measure (CAP-PRI) score	2.0	4.0	3.5	8.0	0.3
Clinically Graded Scale (NCI-CTCAE)	2.0	1.0	2.0	1.0	0.6
*Neurophysiological measurements*					
Sural amplitude (μV)	**3.0**	**7.2**	**7.8**	**9.5**	**0.009**
Tibial amplitude (mV)	**5.7**	**6.8**	**9.9**	**9.6**	**0.01**
*Pain outcome measures*					
Patient-Reported Pain Scale (PNRS)	0	3.0	0	3.0	0.9

### Impact of CIPN severity on sleep quality

Associations between CIPN severity measures and sleep quality of all participants were assessed. Overall, greater CIPN interference on participant’s activities of daily living (PRO-CTCAE) was associated with worse sleep quality (higher PSQI global scores) (*r*_*s*_ = 0.2, *p* = 0.03), including increased reported daytime dysfunction, as assessed by trouble staying awake and problems with keeping up enthusiasm (*r*_*s*_ = 0.3, *p* = 0.002). In addition, the severity of neuropathic pain (PNRS) was also associated with increased reported daytime dysfunction (*r*_*s*_ = 0.2, *p* = 0.03). Furthermore, increased CIPN severity, as assessed on the patient-reported outcome (EORTC-QLQ-CIPN20), was associated with reduced reported hours of sleep (subjective sleep quality subdomain) (*r*_*s*_ = 0.2, *p* = 0.04). However, there were no associations with other CIPN severity outcome measures and sleep parameters, including health-related quality of life (CAP-PRI), clinically graded CIPN (NCI-CTCAE), and neurologically graded CIPN (TNSc, all *p* > 0.05). In order to further examine the impact of CIPN severity on sleep quality, group comparisons between participants with mild CIPN (*n* = 30) and participants with moderate-to-severe CIPN (*n* = 47) were undertaken. Fifty-five percent (*n* = 26) of participants with moderate-severe CIPN attributed their sleep impairments to CIPN symptoms, in comparison to only 10% (*n* = 3) of participants with mild CIPN (*p* < 0.001). Nevertheless, there were no differences in sleep quality between both groups, including all subdomains of the PSQI (all *p* > 0.05).

Similarly, there were no significant differences on CIPN severity measures between participants with poor and good sleep quality. This included the patient-reported outcome measures, the clinically graded scale, and the neurological examination score (all *p* > 0.05) (Table 2). Participants with poor sleep quality had significantly higher sural and tibial amplitudes (both *p* ≤ 0.01) as well as better fine motor skills (Grooved Pegboard Task; *p* = 0.02) than those with good sleep quality, but they were also older than participants with poor sleep quality (Table 1).

### Comparing sleep quality affected by CIPN vs other factors

Participants who reported poor sleep quality (*n* = 58) reported which factors affected their sleep. Overall, more than 50% reported trouble sleeping due to not being able to sleep within 30 min (66%, *n* = 38), waking up in the middle of the night or early morning (88%, *n* = 51), getting up to use the bathroom (64%, *n* = 37), or feeling too hot (55%, *n* = 32) (Supp. Figure 1). Other reasons for sleep disturbance included pain, anxiety, stress, and overthinking (detailed in Supp. Figure 1). However, 41% (*n* = 24) reported that CIPN symptoms were a factor contributing to their poor sleep quality. Participants reported that CIPN-related discomfort or pain led to trouble getting to sleep or caused early waking.

Group comparisons between participants who reported CIPN-induced sleep impairments (*n* = 24) and sleep-impairments due to other factors (*n* = 34) were undertaken (Table 3). More so, overall sleep quality did not differ between both groups (*p* > 0.05), as well as no significant differences in functional assessments or neurophysiological measures between groups (all *p* > 0.05) (Table 4). However, participants with CIPN-induced sleep impairments had significantly higher CIPN severity, including higher scores on patient-reported outcome measures (EORTC-QLQ-CIPN20), HRQoL measure (CAP-PRI), clinically graded scale (NCI-CTCAE), and the neurological examination score (TNSc) (all *p* ≤ 0.01). Furthermore, participants with CIPN-induced sleep impairments had greater perceived CIPN severity (PRO-CTCAE Severity) and greater CIPN interference on activities of daily living (PRO-CTCAE Interference) (both *p* < 0.01) (Table 4).

**Table 3:** Demographic and clinical history of poor sleep quality cohort who had trouble sleeping due to CIPN vs due to other factors. Comparisons between both groups were performed using chi-square tests. Demographic characteristics were compared using independent sample *t*-tests. *Indicates *p*-values using Mann–Whitney *U* tests. *p* < 0.05 was considered significant

Poor sleep quality group (*n* = 58)	Sleep impairments due to other factors (*n* = 34)		CIPN-induced sleep impairments (*n* = 24)		Total (*n* = 58)		*p*-value
	*n*	%	*n*	%	*n*	%	
*Clinical characteristics*							
Female sex	26	77	15	63	41	71	0.3
*Cancer type*							
Breast	3	9	2	8	5	9	0.8
Gynaecological (cervical, endometrial, and ovarian)	10	29	9	38	19	33	
Haematological (myeloma and Hodgkin’s lymphoma)	11	32	4	17	15	26	
GI/colorectal and pancreatic	5	15	5	21	10	17	
Testicular and prostate	1	3	1	4	2	3	
Other	2	6	1	4	3	5	
Missing	2	6	2	8	4	7	
*Chemotherapy type*							
Taxane	18	53	11	46	29	50	0.2
Platinum-based	6	18	9	38	15	26	
Bortezomib	10	29	4	16	14	24	
*Cancer stage*							
I	4	12	2	7	6	10	0.6
II	7	21	3	13	10	18	
III	7	21	8	33	15	26	
IV	3	9	3	13	6	10	
No stage (non-solid tumours)	11	32	4	17	15	26	
Missing	2	5	4	17	6	10	
*Demographic characteristics*							
	Mean	SD	Mean	SD	Mean	SD	*p*-value
Age (years)	61.0	12.2	62.8	8.4	61.7	10.7	0.5
BMI (kg/m^2^)	26.1	5.3	27.1	6.1	26.4	5.5	0.6
Months since treatment completion; median(IQR)*	13.0	20.0	9.0	16.0	12.5	20.0	0.2

**Table 4:** Comparison of sleep outcome measures between participants with trouble sleeping due to CIPN or due to other factors, using Mann–Whitney *U* tests. *p* < 0.05 was considered significant. *Indicates *p*-values using independent sample *t*-tests. Higher scores indicate worse impairment on sleep quality

Poor sleep quality group (*n* = 58)	Sleep impairments due to other factors (*n* = 34)				CIPN-induced sleep impairments (*n* = 24)					*p*-value
	Median			IQR	Median				IQR	
*Functional assessments*										
Grating Orientation Task (GOT) threshold (mm)	3.2			1.3	3.4				1.2	0.1
Average pegboard time (s) (mean, SD)*	69.5			13.7	73.0				16.2	0.4
Von Frey Threshold (mN)	0.2			0.6	0.6				0.6	0.07
*Sleep outcome measures*										
PSQI global score	8.0			4.0	8.0			4.0		0.9
PSQI subjective sleep quality	1.0			1.0	1.5			1.0		0.06
PSQI sleep latency	2.0			2.0	2.0			4.0		0.5
PSQI sleep duration	1.0			1.0	1.0			1.0		0.6
PSQI sleep efficiency (%) (mean, SD)*	74.1			13.2	77.0			16.5		0.5
PSQI sleep disturbance (mean, SD)*	7.2			3.4	8.4			3.6		0.2
PSQI use of sleep medications	0			2.0	0			1.0		0.1
PSQI daytime dysfunction	1.0			1.0	1.0			1.0		0.4
PROMIS sleep disturbance (T-score)	49.1			5.9	52.9			6.2		0.09
*CIPN outcome measures*										
Patient-Reported Outcome (PRO-CTCAE) Severity score		**1.0**	**1.0**			**2.0**	**1.0**			**< 0.001**
Patient-Reported Outcome (PRO-CTCAE) Interference score		**0**	**1.0**			**1.0**	**2.0**			**0.008**
Neurological Examination Score (TNSc)		**3.0**	**4.0**			**5.0**	**3.0**			**0.01**
Patient-Reported Outcome (EORTC-QLQ-CIPN20) score		**10.5**	**10.9**			**22.8**	**11.4**			**< 0.001**
Health-Related Quality of Life Measure (CAP-PRI) score		**1.0**	**4.0**			**6.0**	**6.0**			**< 0.001**
Clinically Graded Scale (NCI-CTCAE)		**1.0**	**1.0**			**2.0**	**0**			**0.001**
*Neurophysiological measures*										
Sural amplitude (μV)		10.5	10.5			7.3	6.2			0.3
Tibial amplitude (mV)		10.3	10.9			9.8	6.7			0.9
*Pain outcome measures*										
Patient-Reported Pain Scale (PNRS)		**0**	**0**			**2.5**	**5.0**			**0.007**

**Fig. 2:**
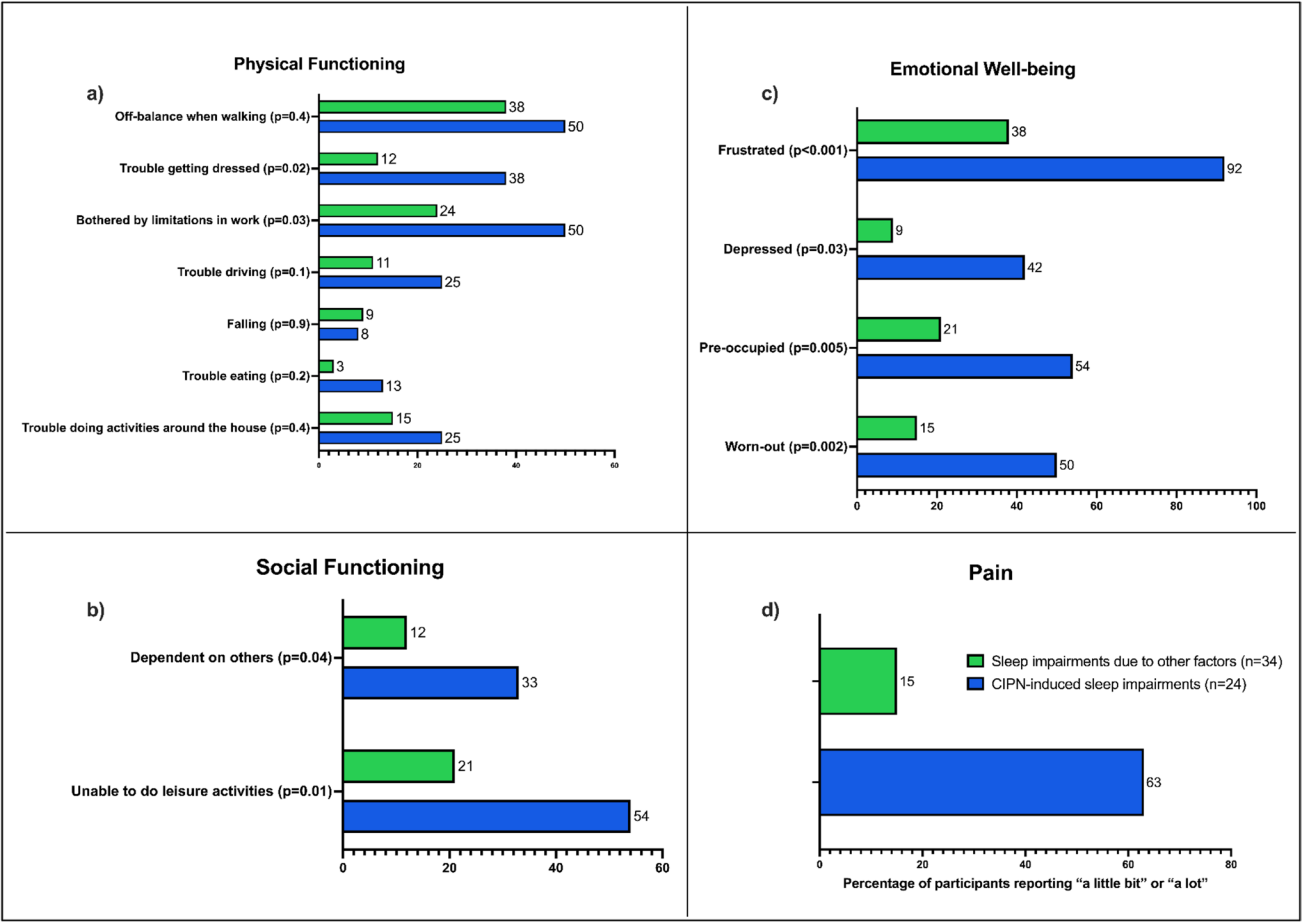
Comparison of percentage of poor sleep quality participants with sleep impairments due to CIPN (*n* = 24) vs other factors (*n* = 34) reporting at least ‘a little bit’ or ‘a lot’ on items of **a** physical functioning, **b** social functioning, **c** emotional well-being, and **d** pain on the HRQoL measure (CAP-PRI), using Mann–Whitney *U* tests. *p* < 0.05 was considered significant

To examine the impact of neuropathy on the quality of life of participants with poor sleep quality, specific items of the HRQoL measure (CAP-PRI) were investigated including physical functioning, social functioning, emotional well-being, and pain (Fig. 2). Overall, participants with CIPN-induced sleep impairments compared to other factors had significantly greater impacts of CIPN on physical functioning, particularly being bothered by limitations in doing work (*p* = 0.03) and trouble getting dressed (*p* = 0.002) (Fig. 2a) as well as greater decline in social functioning, including being dependent on others (*p* = 0.04) and unable to do leisure activities due to their CIPN (*p* = 0.01) (Fig. 2b). They reported being significantly more frustrated, depressed, worn-out, and pre-occupied with their CIPN (all *p* < 0.05) compared to participants with sleep impairments due to other factors (Fig. 2c).

The impact of pain on participants with poor sleep quality was also investigated between both groups. In total, 63% (*n* = 15) of participants with CIPN-induced sleep impairments reported feeling bothered by pain due to CIPN (CAP-PRI Q2), compared to only 15% (*n* = 5) of participants with sleep impairments due to other factors (*p* < 0.001) (Fig. 2d).

## Discussion

This study investigated the sleep quality of neurotoxic chemotherapy-treated patients. Overall, 75% of participants with CIPN reported poor sleep quality, particularly poor subjective sleep quality, increased sleep latency, shorter sleep duration, reduced sleep efficiency, and greater sleep disturbance. Participants reported multiple factors contributing to their poor sleep quality, including difficulty falling asleep, inappropriate waking, getting up to use the bathroom, and temperature disturbance. Importantly, 41% of these participants with poor sleep quality reported CIPN as the cause of their sleep impairments. People with CIPN-induced sleep disturbance reported worse CIPN severity, worse physical and social functioning, and worse emotional well-being and higher incidences of neuropathic pain when compared to participants with sleep impairments attributed to other factors.

Overall, there is a high burden of sleep dysfunction in cancer survivors, even following treatment completion. In our study, three-quarters of participants with CIPN reported poor sleep quality. This is comparable to previous studies on cancer survivors, with percentages ranging from 59 to 80% [3, 13, 29, 30]. There were no differences in CIPN severity between participants with poor sleep quality and participants with good sleep quality. Interestingly, participants with poor sleep quality were younger in age than participants with good sleep quality, in line with previous studies [31, 32]. Although it remains unclear as to why younger patients are at greater risk of developing sleep problems, it could be that they may have better tolerance of treatment, leading to higher doses delivered [33]. They also may have higher levels of psychological distress, which may contribute to a worsening quality of life, in comparison to older patients [34]. However, since our cohort were assessed after chemotherapy treatment completion, the reason for this finding remains unclear.

There have been a number of studies that have investigated sleep quality of cancer patients throughout their chemotherapy treatment, but only a few have investigated the association between sleep quality status and chronic CIPN post-chemotherapy completion. A systematic review and meta-analysis of sleep quality in cancer patients indicated that patients reported poorer sleep quality during their chemotherapy, compared to before commencement and after completion [35]. More so, studies have suggested that patients experience improvements in their overall sleep quality after treatment completion, particularly between 3 and 12 months [36, 37]. However, our study revealed that poor sleep quality persists in neurotoxic chemotherapy-treated patients at a median of 13 months post-neurotoxic chemotherapy.

A limited number of previous studies have described the impact of chronic CIPN on sleep quality, particularly indicating that patients with more severe neuropathy report greater depression, insomnia, and worse health-related quality of life [8, 9]. Our study conducted group comparisons between participants with poor sleep quality due to CIPN versus other factors. In this study, we demonstrated that participants who report poor sleep quality due to CIPN have greater CIPN severity, with significantly more negative impacts on their quality of life, including worse physical, social, and emotional well-being.

Neuropathic pain was also investigated as a potential factor impacting the sleep quality of chemotherapy-treated patients. Among the 7 subdomains of the PSQI, higher incidence of neuropathic pain was significantly associated with daytime dysfunction, which is consistent with previous findings [14]. With a growing body of evidence suggesting that the presence of neuropathic pain may exacerbate poor sleep quality [38], we further investigated patient-reported neuropathic pain in the poor sleep quality cohort and found those with CIPN-induced sleep impairments were substantially more likely to report painful CIPN than those with sleep impairments due to other factors (63% vs 15%, respectively). This finding suggests that chronic painful CIPN adds an additional burden on sleep and quality of life of cancer survivors, which is consistent with previous findings [13].

To date, there have been over 100 tools developed and validated for the assessment of CIPN [39]. This includes a range of patient-reported outcome measures, clinician-based measures, and neurological examination measures, which all aim to assess the severity and degree of CIPN. Unfortunately, the majority of these tools do not contain items designed to assess the presence and severity of sleep problems due to CIPN. Importantly, the most commonly used patient-reported outcome measures for CIPN (reviewed in [40]) do not encompass sleep dysfunction. As evident in the current study, sleep problems due to CIPN exist in a large proportion of neurotoxic chemotherapy-treated cancer survivors. Given that the presence of sleep dysfunction in people with CIPN is associated with worse physical, social, and emotional wellbeing, it is important to utilise tools that recognise sleep dysfunction to identify patient subsets with different clinical characteristics and allow for targeted referral and treatment optimisation.

Overall, this study improves our understanding of the impact of CIPN on the sleep quality of chemotherapy-treated cancer survivors. We utilised well-validated self-reported CIPN outcome measures, as well as a range of objective measures for the assessment of CIPN. Furthermore, we used a validated sleep assessment tool with index cut-offs to identify cohorts with poor sleep quality. Although we used a validated measure, it was self-reported which may introduce bias compared to objective measures of sleep, such as polysomnography [41]. Furthermore, due to the cross-sectional nature of this study, we are limited in our understanding of the progression and impact of CIPN symptoms on sleep quality during treatment. Future prospective studies may examine the development of sleep dysfunction with CIPN, as well as its evolution over time. Given that our cohort was assessed at a median of 13 months post-neurotoxic chemotherapy treatment, additional comorbidities, such as depression, may have developed that could impact sleep; however, they were not examined in our study. Nevertheless, a specific question related to impact of CIPN on sleep was used to categorise participants and attribute the cause of sleep impairment, making it more likely that CIPN was related to sleep dysfunction. In addition, there may be secondary consequences of chronic sleep dysfunction in this population that were not examined, including impact on disease recurrence. Because our study included a mix of cancer and chemotherapy types, this also limits our understanding of the impact of specific cancer and chemotherapy types on overall sleep quality.

## Conclusions

There is a high burden of sleep dysfunction in neurotoxic chemotherapy-treated cancer survivors. These results highlight the persistence and impact of sleep problems due to CIPN long after treatment completion, which contribute to a worsening quality of life. Poor sleep quality was associated with worse CIPN and neuropathic pain, which may impose a great burden on quality of life. Our results reinforce the need to improve the currently used tools to incorporate more focused assessment of sleep quality, which may ultimately help lessen the impact of chronic CIPN on patient function and improve their quality of life.

### Supplementary Information

Below is the link to the electronic supplementary material.

Supplementary file1 (DOCX 6223 KB)

## Data Availability

The datasets generated and analysed during the current study are available from the corresponding author on reasonable request.
