# Supplementary material for: Sleep dysfunction associated with worse chemotherapy-induced peripheral neurotoxicity functional outcomes
Source: Support Care Cancer. 2023 Dec 20;32(1):46. doi: 10.1007/s00520-023-08245-w (PMC10733204; doi:10.1007/s00520-023-08245-w)
Supplement: Supplementary file 1 — Supplementary file1 (DOCX 6223 KB) [file 520_2023_8245_MOESM1_ESM.docx]

**Sleep Dysfunction Associated With Worse Chemotherapy-Induced Peripheral Neurotoxicity Functional Outcomes**

**Fawaz Mayez Mahfouz^1^, Tiffany Li^1^, Masarra Joda^1^, Michelle Harrison^2^, Lisa G. Horvath^2,3,4^, Peter Grimison^2,3^, Tracy King^5,6^, Gavin Marx^7^, David Goldstein^8,9^, Susanna B. Park^1^.**

^1^Brain and Mind Centre, The University of Sydney, Camperdown NSW, 2050, Australia.

^2^Chris O'Brien Lifehouse, Camperdown NSW, 2050, Australia.

^3^Sydney Medical School, The University of Sydney, Camperdown, NSW, 2050, Australia

^4^Royal Prince Alfred Hospital, Camperdown, NSW, 2050, Australia

^5^Cancer Nursing Research Unit, The University of Sydney, Camperdown NSW, 2050, Australia.

^6^Institute of Haematology, Royal Prince Alfred Hospital, Camperdown NSW, 2050, Australia.

^7^Sydney Adventist Hospital, Wahroonga, NSW, 2076, Australia

^8^Prince of Wales Clinical School, Faculty of Medicine & Health, UNSW Sydney, Randwick NSW, 2031, Australia.

^9^Department of Medical Oncology, Prince of Wales Hospital, Randwick NSW, 2031, Australia.

**Journal name:** Supportive Care in Cancer

**Corresponding author:** Susanna B. Park

**E-Mail:** [susanna.park@sydney.edu.au](mailto:susanna.park@sydney.edu.au)

**Supplementary Material**

**Supplementary Methods**

**CIPN and pain assessment: Patient-reported outcome measures**

To assess health-related quality of life, the CAP-PRI included 15 items addressing impact of neuropathy on physical, social, and emotional function. Each item was graded on a 3-point scale comprising of: 0 ‘not at all’; 1 ‘A little bit’; 2 ‘A lot’. A total score was generated, ranging from 0 to 30, with higher scores indicating worse quality-of-life in the 7 days prior to testing [1].

For the assessment of CIPN severity, the validated 20-item patient-reported questionnaire EORTC-QLQ-CIPN20 was used. It assessed peripheral neuropathy symptoms in participants over the 7 days prior to testing. A 4-point Likert scale was used for each item, consisting of the following: 1 ‘Not at all’; 2 ‘A little bit’; 3 ‘Quite a bit’; 4 ‘Very much’. The total score was then converted to a scale ranging from 0 to 100, with higher scores indicating the presence of severe CIPN [2].

The PRO-CTCAE is a 2-item questionnaire. Each item response was scored from 0 to 4, with higher scores reflecting greater severity and interference of neuropathy symptoms in the 7 days prior to testing [3].

The PNRS was used to determine the presence of neuropathic pain. A score of 0 indicated no pain at all, while a score of 10 indicated the worst pain possible in the 7 days prior to testing [4].

**Clinical neuropathy assessment**

Researchers used the NCI-CTCAE to grade participants based on the extent of their CIPN symptoms. The following grades comprise the NCI-CTCAE scale: Grade 0 = no neuropathy symptoms; Grade 1 = asymptomatic, but not interfering with function; Grade 2 = moderate neuropathy symptoms, limiting instrumental activities of daily life (ADL); Grade 3 = severe neuropathy symptoms, limiting self-care ADL; Grade 4 = disabling [5].

The Total Neuropathy Score-clinical version (TNSc©, John Hopkins University) is a composite instrument that comprised the following six domains: patient reported (1) sensory & (2) motor neuropathy symptoms, upper and lower-limb (3) pinprick, (4) vibration, (5) strength and (6) tendon reflex assessments. The grade of each domain ranges from 0 ‘normal’ to 4 ‘severe impairment’ and the sum of these 6 domains ranges from 0 ‘no neuropathy’ to 24 ‘severe neuropathy symptoms’ to generate a neurological examination score [6, 7].

**Functional assessments**

The Grating Orientation Task (GOT) was used to assess sensory perception of participants. The task was conducted on the distal tip of the dominant index finger, using JVP domes with gratings ranging from 0.35 mm to 12 mm in width. The examiner placed the dome with the gratings either vertical or horizonal in a series of 20 random applications. The aim of the task was to identify the smallest grating size that participants could discriminate. A score of at least 15 out of 20 correct was counted as correct identification and subsequent trials were undertaken with a smaller grating size. A GOT threshold was generated according to the scoring protocol. [8].

Upper-limb mechanical detection threshold was measured using the Von Frey monofilaments (Optihair2-Set, Marstock, Nervtest, Germany). A range of monofilament hairs that exert a bending force between 0.125 and 512 millinewtons (mN) were used. The distal tip of the dominant index finger was used for this task, whereby 5 applications were administered. If participants failed to identify 3 out of the 5 applications, then the weight of the monofilament was increased. A total of 5 trials were conducted in a series of increasing and decreasing stimulus intensities, and a final threshold score was generated according to the scoring protocol [9].

The Grooved Pegboard Task was used to assess fine motor skills and manual dexterity. Participants were instructed to place a total of 25 pegs, using their dominant hand only, into grooved holes that are orientated differently along the board. The task was done twice, and a score was calculated in the form of an average time of both trials [10].

**

Supplementary Figure Legend**

**Supplementary Figure 1. Percentage of participants with poor sleep quality (n=58) reporting symptoms on each item of the sleep disturbance subdomain of the PSQI (Q5a to 5j). Percentages are displayed on top of the column graph.**

**References**

1. Gwathmey, K.G., M.R. Conaway, R. Sadjadi, A. Joshi, C. Barnett, V. Bril, E. Ng, W. David, K. Gable, J.T. Guptill, L.D. Hobson-Webb, J. Dineen, M. Hehir, T.H. Brannagan, 3rd, E. Byun, M. Adler, and T.M. Burns, *Construction and validation of the chronic acquired polyneuropathy patient-reported index (CAP-PRI): A disease-specific, health-related quality-of-life instrument.* Muscle Nerve, 2016. **54**(1): p. 9-17.

2. Postma, T.J., N.K. Aaronson, J.J. Heimans, M.J. Muller, J.G. Hildebrand, J.Y. Delattre, K. Hoang-Xuan, M. Lanteri-Minet, R. Grant, R. Huddart, C. Moynihan, J. Maher, R. Lucey, and E.Q.o.L. Group, *The development of an EORTC quality of life questionnaire to assess chemotherapy-induced peripheral neuropathy: the QLQ-CIPN20.* Eur J Cancer, 2005. **41**(8): p. 1135-9.

3. Basch, E., B.B. Reeve, S.A. Mitchell, S.B. Clauser, L.M. Minasian, A.C. Dueck, T.R. Mendoza, J. Hay, T.M. Atkinson, A.P. Abernethy, D.W. Bruner, C.S. Cleeland, J.A. Sloan, R. Chilukuri, P. Baumgartner, A. Denicoff, D. St Germain, A.M. O'Mara, A. Chen, J. Kelaghan, A.V. Bennett, L. Sit, L. Rogak, A. Barz, D.B. Paul, and D. Schrag, *Development of the National Cancer Institute's patient-reported outcomes version of the common terminology criteria for adverse events (PRO-CTCAE).* J Natl Cancer Inst, 2014. **106**(9).

4. Haefeli, M. and A. Elfering, *Pain assessment.* Eur Spine J, 2006. **15 Suppl 1**: p. S17-24.

5. *Common Terminology Criteria for Adverse Events (CTCAE) Version 4.0*. 2010; Available from: <https://evs.nci.nih.gov/ftp1/CTCAE/CTCAE_4.03/CTCAE_4.03_2010-06-14_QuickReference_8.5x11.pdf>.

6. Cornblath, D.R., V. Chaudhry, K. Carter, D. Lee, M. Seysedadr, M. Miernicki, and T. Joh, *Total neuropathy score: validation and reliability study.* Neurology, 1999. **53**(8): p. 1660-4.

7. Cavaletti, G., S. Jann, A. Pace, R. Plasmati, G. Siciliano, C. Briani, D. Cocito, L. Padua, E. Ghiglione, M. Manicone, G. Giussani, and N.G. Italian, *Multi-center assessment of the Total Neuropathy Score for chemotherapy-induced peripheral neurotoxicity.* J Peripher Nerv Syst, 2006. **11**(2): p. 135-41.

8. Van Boven, R.W. and K.O. Johnson, *The limit of tactile spatial resolution in humans: grating orientation discrimination at the lip, tongue, and finger.* Neurology, 1994. **44**(12): p. 2361-6.

9. Rolke, R., R. Baron, C. Maier, T.R. Tolle, D.R. Treede, A. Beyer, A. Binder, N. Birbaumer, F. Birklein, I.C. Botefur, S. Braune, H. Flor, V. Huge, R. Klug, G.B. Landwehrmeyer, W. Magerl, C. Maihofner, C. Rolko, C. Schaub, A. Scherens, T. Sprenger, M. Valet, and B. Wasserka, *Quantitative sensory testing in the German Research Network on Neuropathic Pain (DFNS): standardized protocol and reference values.* Pain, 2006. **123**(3): p. 231-243.

10. Schmidt, S.L., R.M. Oliveira, F.R. Rocha, and Y. Abreu-Villaca, *Influences of handedness and gender on the grooved pegboard test.* Brain Cogn, 2000. **44**(3): p. 445-54.
